# Comparative analysis of complete mitochondrial genomes from *Curcuma longa* and *Curcuma kwangsiensis* reveals structural plasticity, conserved core genes, and species-specific evolutionary dynamics

**DOI:** 10.3389/fpls.2026.1782424

**Published:** 2026-04-15

**Authors:** Yunyi Zhou, Lixiang Yao, Hai Lu, Xueyan Huang, Liying Yu, Chunliu Pan

**Affiliations:** 1 Guangxi Traditional Chinese Medicine (TCM) Resources General Survey and Data Collection Key Laboratory/The Center for Phylogeny and Evolution of Medicinal Plants, National Center for TCM Inheritance and Innovation, Guangxi Botanical Garden of Medicinal Plants, Nanning, China; 2 National Engineering Research Center for Southwest Endangered Medicinal Materials Resources Development, Guangxi Botanical Garden of Medicinal Plants, Nanning, China

**Keywords:** codon usage, *Curcuma kwangsiensis*, *Curcuma longa*, mitochondrial genome, plastid gene transfer, RNA editing, structural dynamics

## Abstract

**Background:**

The Zingiberaceae (ginger family) comprises economically and medicinally important plants, yet mitochondrial genome evolution within this family remains poorly understood. *Curcuma longa* and *Curcuma kwangsiensi*s are widely used medicinal species whose mitogenomic architectures, gene content, and evolutionary trajectories have not been fully characterized. Understanding these features is essential to resolve phylogenetic relationships and elucidate mechanisms of mitochondrial genome plasticity.

**Results:**

We sequenced and assembled the complete mitochondrial genomes of *C*. *longa* and *C*. *kwangsiensis*, revealing highly expanded and fragmented architectures of 7.66 Mb and 7.96 Mb, respectively. Both genomes exhibited conserved GC content (43.7–43.9%) and retained 39 core protein-coding genes, while showing species-specific variation in gene duplication, rRNA/tRNA copy numbers, and the pseudogenization of *sdh3* and *rpl10*. Codon usage analysis indicated a strong A/U bias in synonymous positions, reflecting translational optimization. Extensive RNA editing, predominantly C-to-U conversions, increased hydrophobic residues in membrane-associated proteins, likely enhancing respiratory efficiency. Repeat analysis identified a significantly more robust repeat landscape in *C. kwangsiensis*, which featured 3,059 dispersed repeats in the 300 to 349 bp range and large repeats exceeding 700 bp, whereas repetitive elements in *C. longa* were fewer and predominantly restricted to the first molecule. Sequence divergence analysis revealed that most genes are under strong purifying selection, whereas a limited subset including *atp8*, *ccmFn*, *cox2*, *matR*, *nad4*, *nad7*, and *rpl5* showed episodic positive selection. Whole-genome synteny comparisons indicated profound structural rearrangements and low collinearity, despite high sequence identity within conserved blocks (97.7%). Moreover, both species incorporated 25 plastid-derived fragments, with differences in gene completeness, highlighting ongoing cross-organelle gene transfer. Phylogenetic reconstruction placed both species within a well-supported monophyletic clade with maximum bootstrap support of 100, revealing a sister-taxa relationship between *C. longa* and *C. amarissima* while exhibiting a low substitution rate of 0.004 per site.

**Conclusions:**

Our study demonstrated that *Curcuma* mitogenomes combine remarkable structural plasticity with functional conservation. Conserved core genes, codon usage bias, and extensive RNA editing maintained mitochondrial function, while repetitive sequence proliferation, repeat-mediated recombination, and plastid-derived sequences facilitated lineage-specific diversification despite stable phylogenetic relationships. These findings provide essential genomic resources for the genus *Curcuma* and offer insights into mitochondrial genome evolution and species-specific diversification in Zingiberaceae.

## Introduction

The family Zingiberaceae, commonly known as the ginger family, is a vital group of monocotyledonous plants comprising approximately 50 genera and over 1,600 species. This family is renowned for its immense economic, medicinal, and ornamental value (Kress et al., 2002). Among its most significant genera is *Curcuma* L., which is widely distributed throughout tropical and subtropical Asia. The type species of this genus, *Curcuma longa* L. (turmeric), is globally cultivated for its rhizomes, which are used as a spice, a food coloring agent, and a key component in traditional medicine systems, largely due to the presence of bioactive curcuminoids (Hewlings and Kalman, 2017; Priyadarsini, 2014). Similarly, *Curcuma kwangsiensis* is another medicinally important species, utilized in traditional Chinese medicine for its purported anti-inflammatory and anti-cancer properties (Li et al., 2010). Despite their widespread use and economic importance, the phylogenetic relationships within the genus *Curcuma* are complex and not yet fully resolved, often hampered by morphological plasticity and interspecific hybridization (Záveská et al., 2012). This highlights the urgent need for robust molecular data to clarify species boundaries and evolutionary history.

Molecular systematics of the Zingiberaceae has long faced challenges due to frequent incongruence between morphological classifications and molecular phylogenies. In one of the earliest large-scale molecular studies, Kress et al. (2002) used *matK* and nuclear ITS sequences to reconstruct the phylogeny of the family and proposed a revised tribal and generic classification (Kress et al., 2002). Their results revealed that several morphologically defined tribes and genera were not monophyletic under molecular evidence, and that the relationships among *Curcuma* and its allied genera were weakly supported or topologically unstable. Subsequent studies with expanded sampling at the generic level, such as Kress et al. (2005) on *Alpinia*, further confirmed that traditional morphological features (e.g., inflorescence structure) are often homoplasious and that both plastid and nuclear markers may fail to fully resolve deep or shallow relationships within the family (Kress et al., 2005). Focusing on *Curcuma*, Záveská et al. (2012) analyzed combined plastid (*matK*, *psbA-trnH*, *trnL-trnF*) and nuclear ITS datasets, and found pronounced topological incongruence between plastid and nuclear phylogenies (Záveská et al., 2012). They attributed these conflicts to complex evolutionary processes such as polyploidy, historical hybridization, and incomplete lineage sorting, all of which obscure species boundaries and undermine phylogenetic resolution. More recently, Skopalíková et al. (2023) provided genomic-scale evidence of ancient hybridization events in *Curcuma*, reinforcing the view that the genus has experienced a reticulate evolutionary history that single or few-locus markers cannot disentangle (Skopalíková et al., 2023). Collectively, these studies illustrate that both traditional morphological traits and commonly used plastid or nuclear loci (e.g., *matK*, ITS) provide insufficient resolution to reconstruct a stable phylogeny of *Curcuma* and its relatives.

The plant mitochondrial genome (mitogenome) is one of the three genomes found in plant cells, playing a crucial role in cellular respiration and energy production (Logan, 2006). In contrast to the relatively compact and conserved chloroplast genomes, plant mitogenomes are typically much larger and exhibit extraordinary structural plasticity, with significant variations in size, gene order, and repeat content even among closely related species (Gualberto and Newton, 2017). This structural dynamism is largely attributed to frequent recombination events mediated by repetitive sequences (Christensen, 2018). Conversely, the protein-coding sequences within plant mitogenomes evolve at a much slower rate compared to those in chloroplast and nuclear genomes (Wolfe et al., 1987). This unique combination of structural liability and sequence conservatism makes the mitogenome a powerful tool for investigating both deep-level phylogenetic relationships and micro-evolutionary processes such as genome rearrangement and adaptation (Kozik et al., 2019). However, despite the growing recognition of mitogenomes as a complementary and independent source of evolutionary information, mitochondrial genome research within this family remains notably limited. To date, only a few mitochondrial genomes from the ginger family have been reported, such as those of *C. amarissima* and *C. longa* (Liang et al., 2025; Xu et al., 2026), but a comprehensive comparative analysis within the genus *Curcuma* is still lacking. Specifically, the complete mitogenome of *C*. *kwangsiensis* and *C*. *longa* remains uncharacterized. This knowledge gap hinders a deeper understanding of mitogenome evolution, structural variation, and the precise phylogenetic placement of these species within the Zingiberaceae.

In this study, we sequenced, assembled, and annotated the complete mitochondrial genomes of *C. longa* and *C. kwangsiensis*. The primary objectives of our research were: (1) to characterize and compare the genomic features of these two mitogenomes, including genome size, gene content, codon usage, and repeat sequences; (2) to investigate genomic structural variations, such as gene rearrangements and syntenic relationships, between the two *Curcuma* species and other available Zingiberaceae mitogenomes; and (3) to reconstruct a robust phylogenetic tree based on mitochondrial protein-coding genes to accurately determine the evolutionary positions of *C. longa* and *C. kwangsiensis*. The results of this study will provide valuable genomic resources for the *Curcuma* genus and offer new insights into the evolutionary dynamics of mitogenomes within the ginger family.

## Materials and methods

### Plant material, DNA extraction, and sequencing

Fresh young leaves of *Curcuma longa* and *Curcuma kwangsiensis* were collected from the plant cultivation research base of the Guangxi Botanical Garden of Medicinal Plants (Nanning, Guangxi, China). Voucher specimens were identified by Prof. Liying Yu. Total genomic DNA was extracted from the leaves using a modified CTAB method. The quality and quantity of the extracted DNA were assessed using a NanoDrop 2000 spectrophotometer and 1.0% agarose gel electrophoresis.

A combination of sequencing platforms was used to generate the genomic data. High-molecular-weight DNA was used for long-read sequencing on the Nanopore PromethION platform, and 24.88 Gb raw data with average reads size of 780418 bp were produced in *C. longa*, 23.08 Gb raw data with average reads size of 1068876 bp were produced in *C. kwangsiensis* (**Supplementary Table 8**). Data were then filtered and re-edited using NanoFilt and NanoPlot in Nanopack software (De Coster et al., 2018). A total of 22.65 Gb data were generated from 2.92 million reads with average reads size of 77.65 kb in *C. longa*, and 20.34 Gb data were generated from 1.90 million reads with average reads size of 106.90 kb in *C. kwangsiensis* (**Supplementary Table 8**). Meanwhile, the libraries with average fragment length of 350 bp were constructed with the high-quality DNA using the NexteraXT DNA Library Preparation Kit. Sequencing was then performed on the Illumina Novaseq 6000 platform, generating 150 bp paired-end reads for genome survey and error correction. 24.11 Gb and 22.20 Gb raw sequence data were produced in *C. longa* and *C. kwangsiensis*, respectively (**Supplementary Table 8**). After edition using the NGS QC Tool Kit v2.3.3 (Patel and Jain, 2012), 23.63 Gb data were generated from 159.45 million clean reads in *C. longa*, 21.72 Gb data were generated from 146.73 million clean reads in *C. kwangsiensis* (**Supplementary Table 8**).

### Mitochondrial genome assembly and annotation

The mitochondrial genomes were assembled using a hybrid strategy combining Oxford Nanopore long-read and Illumina short-read data. To enrich for mitochondrial sequences, raw Nanopore reads were initially aligned to the reference mitogenome of *Curcuma amarissima* (NCBI: PQ442956.1-PQ442994.1) using minimap2 (v2.15-r905). The filtered reads were then subjected to *de novo* assembly using miniasm (v0.3-r179) to generate initial scaffolds. To ensure high sequence accuracy, the initial assembly was polished with Illumina short-read data using NextPolish (v1.3.1, https://github.com/Nextomics/NextPolish). To further validate the structural integrity of the expanded mitogenomes, Illumina data were independently assembled into contigs using Unicycler (v0.4.8). These high-fidelity contigs were visualized using Bandage (v0.8.1) and served as structural anchors (Wick et al., 2017, Wick et al., 2015). The final assembly was achieved by aligning the corrected Nanopore scaffolds with the Unicycler-derived contigs using minimap2, followed by manual refinement to resolve complex repeat regions and confirm scaffold connectivity. This hybrid approach ensured both the structural completeness of the large mitogenomes and high nucleotide-level accuracy. A complete mitochondrial genome of *C. longa* and *C. kwangsiensis* was obtained, respectively. The complete mitogenome was annotated by MITOFY and MFANNOT (Alverson et al., 2010; Gautheret and Lambert, 2001). The map of the mitogenome was visualized using the OGDRAW program (Greiner et al., 2019). Finally, the mitochondrial genome datasets of *C. longa* and *C. kwangsiensis* with accession number PX734729, PX734730, PX734731, PX734732, PX754766, PX754767, PX754768, PX754769 and PX754770 ave been deposited in the GenBank of NCBI, respectively.

### Repeat sequence analysis

Repeat sequences were identified using multiple approaches. Microsatellites (SSRs) were detected using MISA v1.0 (Beier et al., 2017) with the parameter string “1-10 2-5 3-4 4-3 5-3 6-3”. Tandem repeats were identified using Tandem Repeats Finder (TRF) v4.09 (Benson, 1999) with parameters of 2, 7, 7, 80, 10, 50, and 2000, along with the -f, -d, and -m flags. Dispersed repeats, including forward, reverse, and palindromic repeats, were identified using the online REPuter server (https://bibiserv.cebitec.uni-bielefeld.de/reputer/) with a minimal repeat size set to 275 bp. The distribution and types of repeats were visualized using Circos v0.69-6 (Zhang et al., 2013).

### Codon usage analysis

Protein-coding genes (PCGs) longer than 300 bp, starting with an ATG codon and ending with a TAA, TAG, or TGA stop codon, were extracted from the genomes. The Relative Synonymous Codon Usage (RSCU) values for all PCGs were calculated using CodonW v1.4.4.

### Prediction of RNA editing sites

Potential RNA editing sites (C-to-U conversions) in the PCGs of both mitogenomes were predicted using the Plant mitochondrial RNA editing prediction (PmtREP) tool with default settings.

### Homologous DNA transfer analysis

To identify mitochondrial insertions of plastid DNA, the mitogenomes of *C. longa* and *C. kwangsiensis* were compared against their respective chloroplast genomes using BLASTn v2.9.0+ (Altschul et al., 1990). The analysis was conducted with an E-value cutoff of 1e-5 and a word size of 7. Homologous fragments exceeding 1,000 bp were retained, and their genomic locations were visualized using Circos v0.69-6 (Zhang et al., 2013).

### Gene loss analysis

The presence, absence, pseudogenization, and copy number of mitochondrial genes across the two *Curcuma* species and other selected Zingiberaceae species were compared. The resulting gene distribution matrix was visualized as a heatmap using R v3.6.0.

### Synteny analysis

Pairwise synteny between *C. longa* and *C. kwangsiensis*, as well as comparisons with other related species, were conducted. Homologous regions were first identified using BLASTn v2.9.0+ (Altschul et al., 1990) with an E-value cutoff of 1e-5 and a word size of 7. Fragments larger than 1,000 bp were defined as conserved collinear blocks and visualized as a multiple synteny plot using TBtools v2.119 (Chen et al., 2020).

### Sequence divergence and selective pressure

To assess sequence divergence, the 29 shared homologous genes between *C. longa* and *C. kwangsiensis* were aligned using MAFFT v7.429 (Katoh and Standley, 2013). The nucleotide diversity (Pi) for each gene was then calculated using DnaSP6 (Rozas et al., 2017) with a 200 bp window and a 100 bp step size.

To analyze selective pressure, orthologous gene pairs from all compared species were extracted. Protein-coding DNA alignments were constructed using ParaAT2.0 (Zhang et al., 2012). The non-synonymous (Ka) and synonymous (Ks) substitution rates were subsequently calculated using KaKs_Calculator v2.0 (https://sourceforge.net/projects/kakscalculator2/) employing the YN method.

### Phylogenetic analysis

Phylogenetic analysis was conducted using both mitochondrial and chloroplast genomes from the two newly sequenced *Curcuma* species and 16 other species from GenBank. OrthoFinder v2.3.14 (Emms and Kelly, 2019) was used to identify single-copy orthologous genes among all species. This process yielded 14 shared mitochondrial genes including *atp6*, *ccmB*, *ccmC*, *ccmFc*, *cob*, *cox2*, *nad1*, *nad2*, *nad4*, *nad4L*, *nad5*, *nad6*, *nad7*, and *rps12*. For the chloroplast phylogeny, 47 single-copy orthologous genes were identified. These genes included *accD*, *atpA*, *atpB*, *atpE*, *atpF*, *atpH*, *atpI*, *clpP*, *petA*, *petG*, *petN*, *psaA*, *psaB*, *psaI*, *psaJ*, *psbA*, *psbB*, *psbC*, *psbD*, *psbE*, *psbF*, *psbH*, *psbK*, *psbL*, *psbM*, *psbN*, *psbT*, *psbZ*, *rbcL*, *rpl14*, *rpl20*, *rpl22*, *rpl32*, *rpl33*, *rpl36*, *rpoA*, *rpoB*, *rpoC1*, *rpoC2*, *rps11*, *rps14*, *rps18*, *rps3*, *rps4*, *rps8*, *ycf3*, and *ycf4*.

Mitochondrial sequences were aligned individually using MAFFT v7.429 (Katoh and Standley, 2013). Chloroplast sequences were aligned using MAFFT v7.525 based on codon mode. Ambiguously aligned regions were trimmed using Gblocks 0.91b (Talavera and Castresana, 2007) to remove positions containing gaps with the -t=c parameter. The trimmed alignments were then concatenated into separate supermatrices for tree inference. Maximum Likelihood (ML) phylogenetic trees were inferred using IQ-TREE (Nguyen et al., 2015). Mitochondrial data used version 1.6.12 while chloroplast data used version 3.0.1. Best-fit nucleotide substitution models were determined by the integrated ModelFinder based on the Bayesian Information Criterion (BIC). The TIM+F+R2 and GTR+F+I+R2 models were selected for the mitochondrial and chloroplast datasets, respectively. Node support was assessed using an Ultrafast bootstrap analysis with 1,000 replicates. *Phoenix dactylifera* (NC013991) was used as the outgroup to root the phylogeny.

## Result

### Mitochondrial genome assembly and annotation

**Figure 1:**
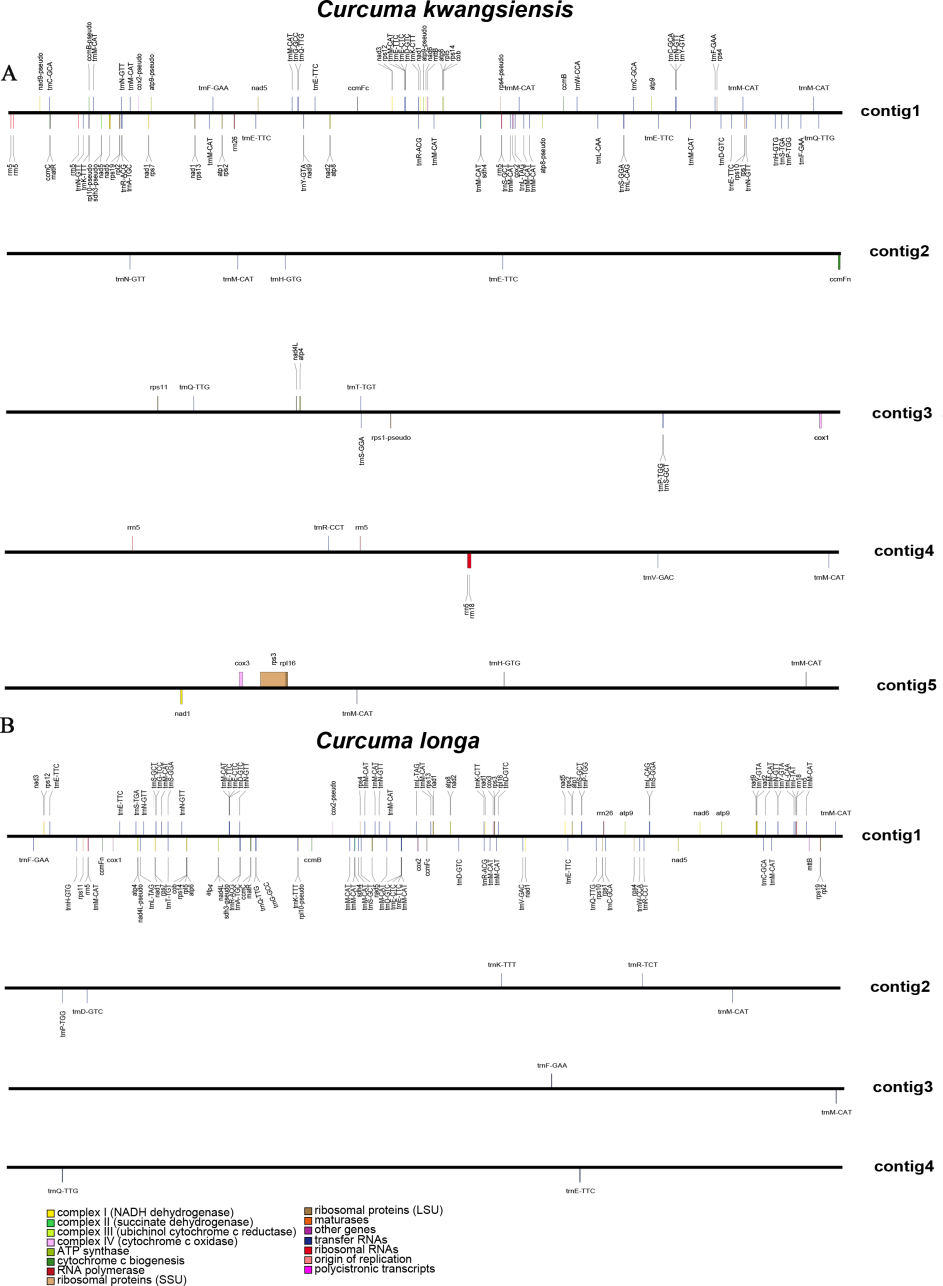
Mitogenome map of *C. kwangsiensis***(A)** and *C. longa***(B)**.

The mitochondrial genomes of *C. kwangsiensis* and *C. longa* were assembled into five and four linear scaffolds (**Figures 1A, B**), with total genome sizes of 7.96 Mb and 7.66 Mb, respectively (**Table 1**). Visualization of the assembly paths using Bandage revealed a complex, interconnected network (**Supplementary Figure 1**), indicating that these scaffolds represented a highly rearranged physical structure. To validate the authenticity of these unusually expanded mitogenomes and rule out nuclear DNA contamination, we performed a sequencing depth analysis. As shown in the assembly graph, all major contigs exhibited high and remarkably uniform sequencing coverage. Furthermore, the sequencing depth distribution analysis confirmed a robust and consistent average coverage of 104.0× across the entire 7.96 Mb in the *C. kwangsiensis* genome, and 244.8× across the entire 7.66 Mb in the *C. longa* genome the sequencing depth distribution analysis confirmed a robust and consistent average coverage of 104.0× across the entire 7.96 Mb in the *C. kwangsiensis* genome, and 244.8× across the entire 7.66 Mb in the *C. longa* genome (**Supplementary Figure 2**), providing additional support for the mitochondrial origin of the sequence. This consistent depth significantly exceeded the anticipated background nuclear coverage, providing robust evidence that these huge sequences were of authentic mitochondrial origin. Our findings demonstrated that both *Curcuma* species possess exceptionally large mitogenomes, representing some of the most significantly expanded architectures reported in angiosperms to date.

**Table 1:** Assembly statistics for the *C. kwangsiensis* and *C. longa* mitogenome genome.

Species	Contig	Genome			CDS			tRNA			rRNA		
		Type	Length	GC(%)	Number	Length	GC(%)	Number	Length	GC(%)	Number	Length	GC(%)
*Curcuma kwangsiensis*	1	linear	5847369	43.9	30	24708	43.11	51	3787	50.49	5	4780	49.96
	2	linear	704359	43.86	1	1710	46.96	4	286	51.75	–	–	–
	3	linear	691005	43.56	6	5736	43.11	5	351	52.42	–	–	–
	4	linear	532004	44.42	–	–	–	3	213	47.42	4	2469	52.65
	5	linear	181266	43.32	4	3972	43.63	3	214	46.73	–	–	–
*Curcuma longa*	1	linear	7032569	43.69	42	35256	43.26	65	4852	50.54	4	6366	51.23
	2	linear	421372	43.49	–	–	–	5	344	49.13	–	–	–
	3	linear	112178	44.1	–	–	–	2	146	45.21	–	–	–
	4	linear	89843	44.96	–	–	–	2	144	48.61	–	–	–

The GC content was nearly identical between the two species, reaching 43.90% in *C. kwangsiensis* and 43.69% in *C. longa*, indicating a similar compositional bias. The CDS regions spanned 24.71 kb in *C. kwangsiensis* and 35.26 kb in *C. longa*, and both species exhibited a similar GC level within coding regions, averaging around 44.11% and 43.26%, respectively. A total of 114 functional genes were identified in *C. kwangsiensis*, whereas *C. longa* encoded 120 functional genes (**Supplementary Table 1**). Although *C. kwangsiensis* contained fewer CDS and tRNA copies, with 39 protein-coding genes and 66 tRNAs, compared with 42 CDS and 74 tRNAs in *C. longa*. Both species shared the same set of 39 conserved protein-coding genes, including nine *nad* genes (complex I), five *atp* genes (complex V), three *cox* genes (complex IV), one *cob* gene (complex III), four *ccm* genes associated with cytochrome c biogenesis, fourteen ribosomal protein genes from both the large and small subunits, one *sdh4*, one *matR*, and one *mttB*. Additionally, *nad1* was fragmented across separate scaffolds in *C. kwangsiensis*, whereas the corresponding gene remained more structurally continuous in *C. longa*, indicating species-specific genome rearrangement patterns. Differences were also detected in rRNA gene composition. *C. kwangsiensis* contained nine rRNA genes, including seven copies of *rrn5*, whereas *C. longa* encoded only four rRNA genes, with two *rrn5* copies. Despite variation in copy number, both species retained one *rrn18* and one *rrn26*.

**Figure 2:**
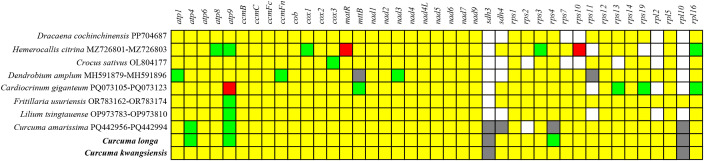
The PCGs composition of *C. kwangsiensis* and *C. longa* mitogenomes. The composition of PCGs in the mitogenomes of ten species. Yellow, green and red boxes indicated that one, two and more copies in genomes, respectively. White boxes indicated that the gene was absent in genomes. Grey boxes indicated that the gene was pseudogene.

To assess the protein-coding genes (PCGs) integrity of the *C. kwangsiensis* and *C. longa* mitochondrial genomes, a comparative analysis of 41 core PCGs was conducted against several related species (**Figure 2**). Overall, a highly conserved gene composition was observed within both *Curcuma* species. Among 41 PCGs, *sdh3* and *rpl10* were pseudogenized in both species, suggesting potential gene loss events that occurred prior to species divergence. However, *C. longa* displayed increased gene redundancy, with additional duplicated copies of *atp4*, *atp9*, and *rps4* that were retained as single-copy genes in *C. kwangsiensis*. Overall, the comparison highlights a conserved core mitochondrial gene repertoire in *Curcuma*, accompanied by moderate divergence in gene duplication, rRNA expansion, and structural fragmentation.

### Codon usage

**Figure 3:**
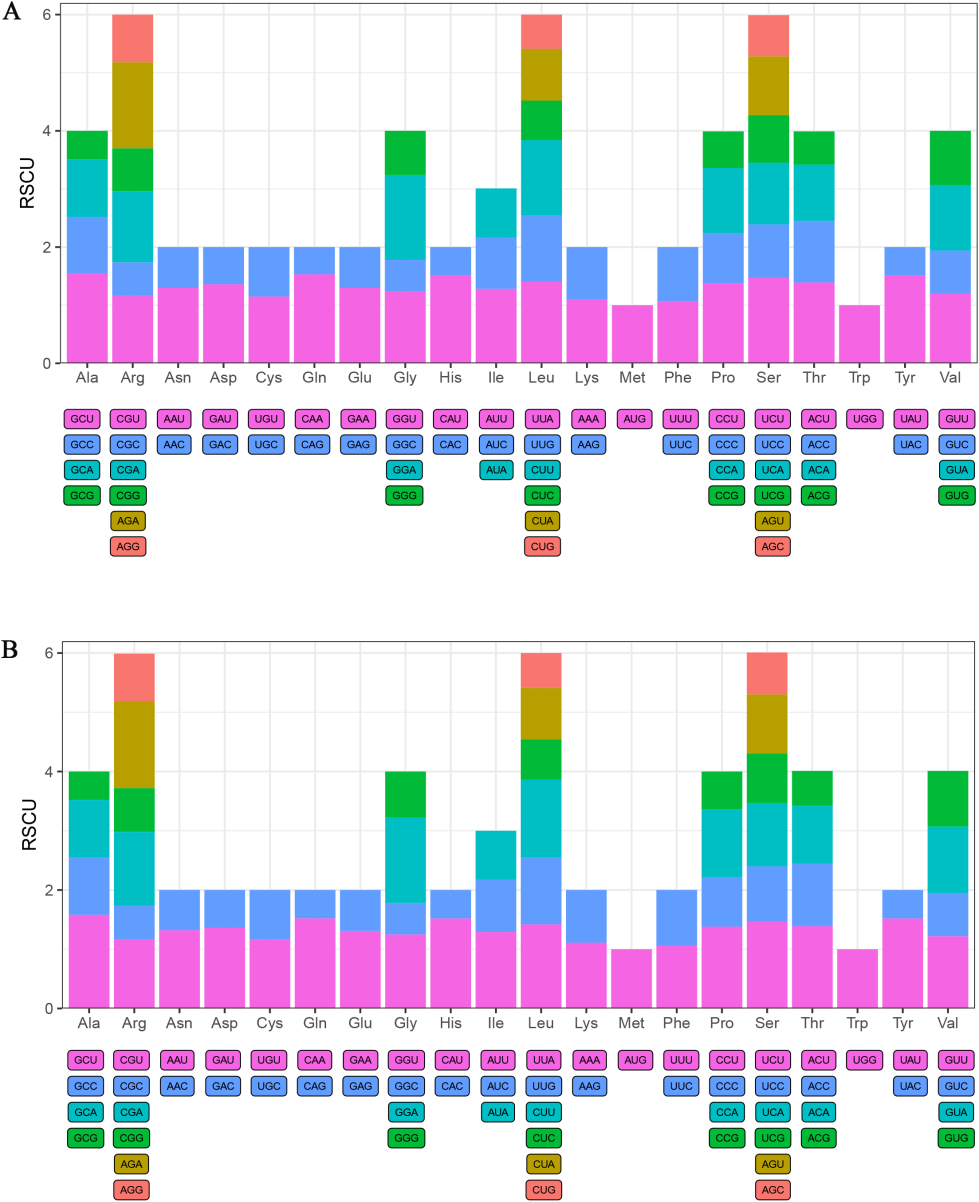
Relative Synonymous Codon Usage (RSCU) in the protein-coding genes of *C. kwangsiensis***(A)** and *C. longa***(B)** mitogenomes. Codons with an RSCU value greater than 1.0 are considered preferred codons.

The codon usage patterns of the protein-coding genes (PCGs) in the *C. longa* and *C. kwangsiensis* mitogenomes were analyzed (**Figure 3**, **Supplementary Table 2**). The PCGs of *C. kwangsiensis* utilized a total of 9770 codons, while those of *C. longa* utilized 9,879 codons (**Supplementary Table 2**). The amino acid compositions derived from these codons were highly conserved between the two species. Leucine (Leu) was the most abundant amino acid in both mitogenomes, constituting 10.4% of all codons in both species and representing 1,013 codons in *C. kwangsiensis* and 1,023 codons in *C. longa*. Serine (Ser) was the second most frequent, comprising approximately 9.2% of the codons in both species. Conversely, Tryptophan (Trp) and Cysteine (Cys) were the least frequently encoded amino acids, each accounting for only about 1.4–1.5% of the total. The canonical AUG codon served as the primary start codon for methionine. All three standard termination codons (UAA, UAG, and UGA) were identified in the PCGs of both mitogenomes, with UAA stop codon being the most frequently used.

RSCU analysis was performed to determine codon usage bias. A strong and consistent bias toward codons ending in Adenine (A) or Uracil (U) was observed in both species. Excluding methionine (Met) and tryptophan (Trp), which are encoded by single codons, the remaining 18 amino acids with synonymous codons, all showed a clear preference (RSCU > 1) for codons ending with A or U over those ending with Cytosine (C) or Guanine (G). A virtually identical hierarchy was observed in *C. kwangsiensis*, where the GCU (Alanine) codon’s RSCU value was 1.54, and the CAA (Glutamine) codon’s value was 1.53 (**Figure 3A**). In *C. longa*, the GCU codon for Alanine showed the highest usage bias, with an RSCU value of 1.58. This was closely followed by UAU (Tyrosine), CAU (Histidine), and CAA (Glutamine), all displaying high RSCU values of 1.52 (**Figure 3B**). Conversely, their synonymous counterparts ending in G or C were strongly under-represented (RSCU < 1). For instance, the GCG (Alanine) and UAC (Tyrosine) codons both had RSCU values between 0.48–0.49. This indicates a significant A/T bias in the third codon position, a common feature in angiosperm mitogenomes. Overall, the amino acid frequencies, RSCU values, and codon usage patterns were nearly identical between *C. kwangsiensis* and *C. longa*, suggesting highly conserved translational dynamics and evolutionary pressure within the *Curcuma* genus.

### Repeat sequences

**Figure 4:**
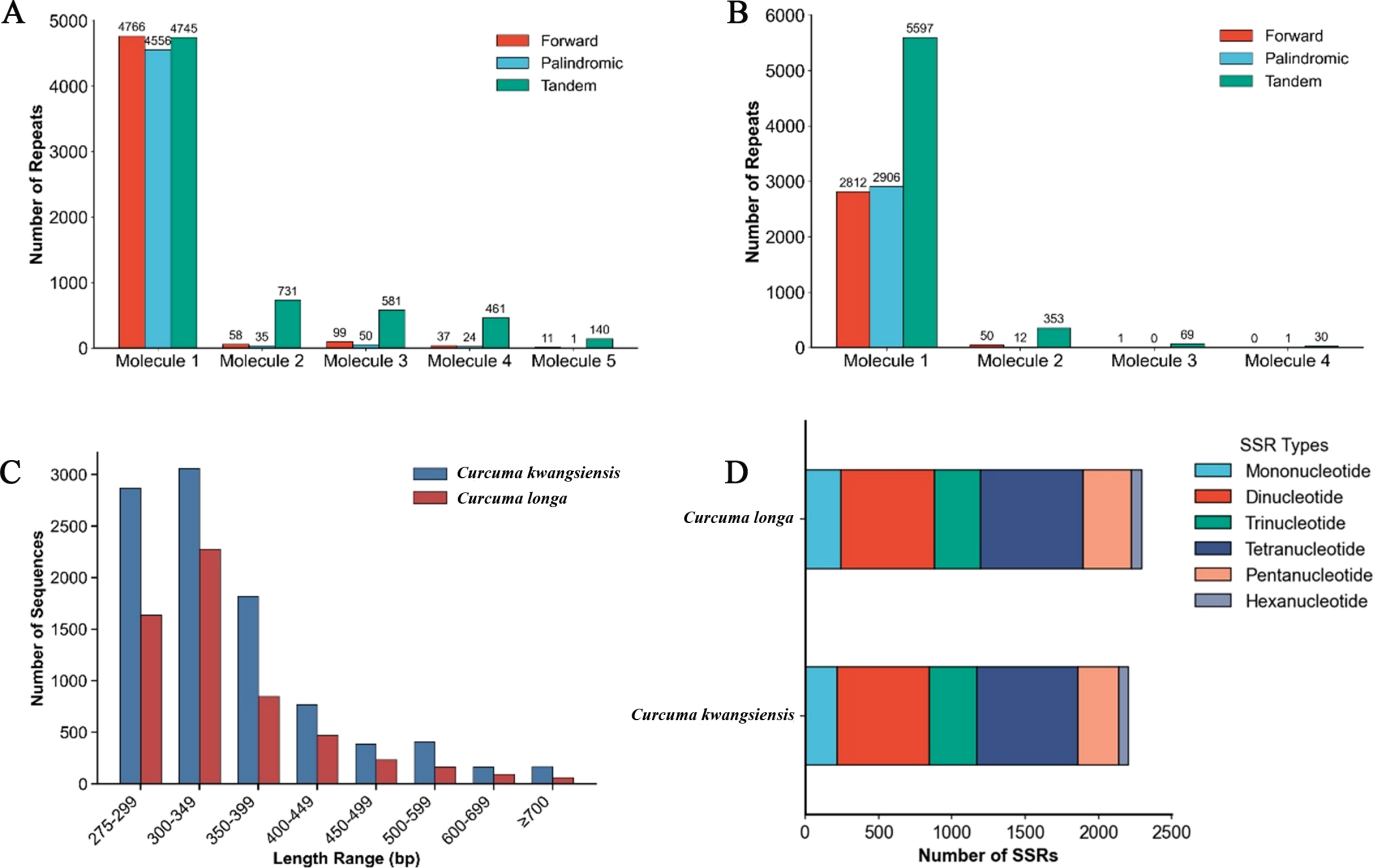
Repeat sequence and simple sequence repeats in the mitogenomes of *C. longa* and *C. kwangsiensis*. **(A)**, number and types of repeats including forward, palindromic, and tandem sequences distributed across the five molecules of the *C. kwangsiensis* mitogenome. **(B)**, distribution of repeat types across the four molecules of the *C. longa* mitogenome. **(C)**, comparative frequency distribution of repeat sequences categorized by length ranges. **(D)**, total number and composition of Simple Sequence Repeat (SSR) types ranging from mononucleotide to hexanucleotide motifs for both *Curcuma* species.

Comparative analysis of the mitogenomes of *C. kwangsiensis* and *C. longa* revealed a substantial number of dispersed repeats, which are pivotal in mediating genomic rearrangements. Overall, the total number of dispersed repeats in *C. kwangsiensis* significantly exceeded those in *C. longa* (**Figures 4A, B**). Intriguingly, only forward (F) and palindromic (P) repeats were observed across both mitogenomes, which suggests these specific repeat types play a dominant role in driving the structural evolution and potential multipartite configuration of *Curcuma* mitogenomes. In both species, molecule 1 harbored the vast majority of these repeats, and *C. kwangsiensis* showed a much more robust repeat landscape than those in *C. longa*. The length of these repeats predominantly ranged from 275 to 499 bp, with a distinct peak observed in the 300–349 bp, comprising 3,059 and 2,277 repeats for *C. kwangsiensis* and *C. longa*, respectively (**Figure 4C**). The frequency of repeats exhibited a sharp decline as sequence length increased. Notably, in the category of large repeats (≥700 bp), *C. kwangsiensis* maintained a substantially higher abundance (169 repeats) than *C. longa* (60 repeats), potentially contributing to the larger genome size and higher complexity of the former.

In terms of tandem repeats, *C. kwangsiensis* exhibited a notably higher frequency and greater total count than *C. longa*, further distinguishing the repetitive landscapes of the two species (**Supplementary Table 3**). A notable presence across all five molecules in *C. kwangsiensis*, while tandem repeats in *C. longa* were predominantly restricted to the first molecule, with significantly lower counts in molecules 2 through 4. Tandem repeats were identified with varying consensus lengths and copy numbers, with repeat units ranging from 2 bp to 685 bp in *C. longa* and extending from 2 bp to a maximum of 861 bp in *C. kwangsiensis* (**Supplementary Table 3**). The sequence identity for these tandem repeats remained remarkably high, generally ranging from 70% to 100%, indicating that these sequences may have originated from relatively recent duplication events. Tandem repeats were found to be ubiquitous throughout the mitogenomes, contributing further to the expansion of genome size and the promotion of genetic diversity.

Simple sequence repeats (SSRs) are essential molecular features that significantly influence genome evolution and stability, and our systematic survey revealed a higher density of SSRs in *C. kwangsiensis* compared to *C. longa* (**Figure 4D**). In both mitogenomes, SSRs were primarily composed of tetranucleotide and dinucleotide motifs, with hexanucleotide repeats being the least. Genomic localization analysis indicated that these SSRs are predominantly situated in the IGS. In *C. kwangsiensis*, SSRs were identified within the coding sequences of four genes, namely *ccmC*, *matR*, *cob*, and *rps3*, whereas *C. longa* featured an almost identical distribution profile, differing only by the inclusion of a single additional gene, *atp9* (**Supplementary Table 3**). The high degree of polymorphism observed in SSR motifs between the two species highlights their potential utility as molecular markers for population genetic studies and species identification within the *Curcuma* genus.

### RNA editing sites

**Figure 5:**
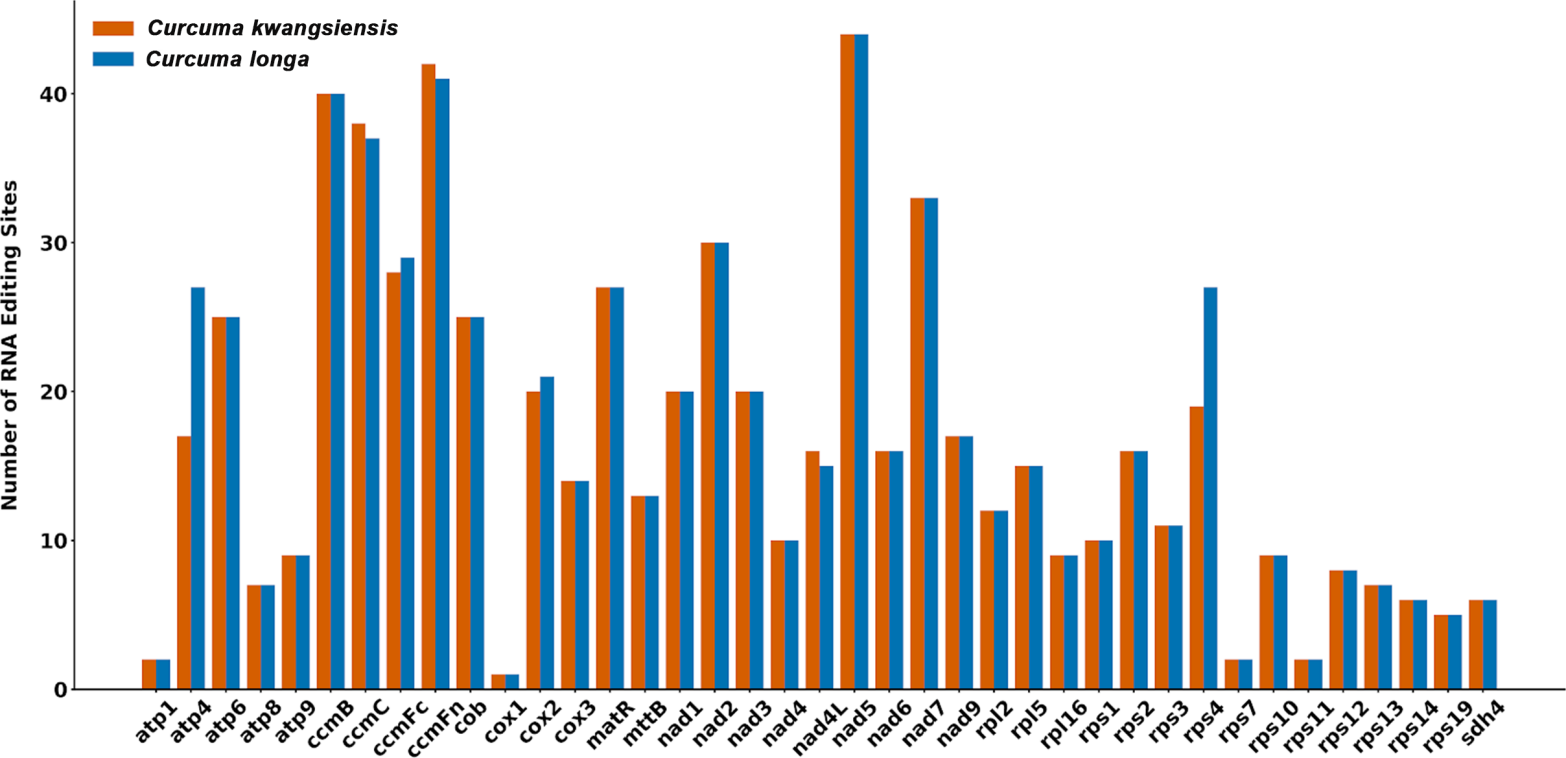
Number of predicted RNA editing sites in the 39 shared protein-coding genes of *Curcuma kwangsiensis* and *Curcuma longa* mitogenomes.

RNA editing sites were predicted across the 39 protein-coding genes (PCGs) in both *Curcuma* mitogenomes (**Figure 5**). A total of 668 RNA editing sites were identified in *C. longa*, while a slightly lower number, 651, were found in *C. kwangsiensis*. These edits resulted in 32 types of amino acid substitutions (**Supplementary Table 4**). The most frequent substitutions observed in both *C. kwangsiensis* and *C. longa* mitogenomes were Serine to Leucine, representing 11.98% of total RNA editing sites. Functionally, the vast majority of these edits converted hydrophilic residues to hydrophobic ones, a pattern conserved in both species, with *C. kwangsiensis* showing 519 sites comprising 79.72% of its substitutions, and *C. longa* showing 533 such conversions representing 79.79% of its total (**Table 2**). This suggests a strong selective pressure to increase protein hydrophobicity via RNA editing. Additionally, 7 editing events were found to create new stop codons.

**Table 2:** The type of RNA editing sites in *C. kwangsiensis* and *C. longa* mitogenome genome.

Type	RNA-editing	*C. kwangsiensis*	*C. longa*
hydrophilic-hydrophilic	CAC (H) => TAC (Y)	9	9
	CAT (H) => TAT (Y)	23	24
	CCA (P) => TCA (S)	10	11
	CCC (P) => TCC (S)	14	15
	CCG (P) => TCG (S)	6	5
	CCT (P) => TCT (S)	27	27
	CGC (R) => TGC (C)	8	8
	CGT (R) => TGT (C)	28	29
	total	125	128
hydrophilic-hydrophobic	ACA (T) => ATA (I)	7	8
	ACC (T) => ATC (I)	12	11
	ACG (T) => ATG (M)	11	11
	ACT (T) => ATT (I)	9	10
	CCA (P) => CTA (L)	43	45
	CCC (P) => CTC (L)	12	13
	CCC (P) => TTC (F)	6	6
	CCG (P) => CTG (L)	33	34
	CCT (P) => CTT (L)	24	25
	CCT (P) => TTT (F)	12	12
	CGG (R) => TGG (W)	37	37
	CTC (L) => TTC (F)	17	18
	CTT (L) => TTT (F)	32	33
	GCA (A) => GTA (V)	10	10
	GCC (A) => GTC (V)	4	4
	GCG (A) => GTG (V)	8	8
	GCT (A) => GTT (V)	7	7
	TCA (S) => TTA (L)	78	80
	TCC (S) => TTC (F)	38	39
	TCG (S) => TTG (L)	52	54
	TCT (S) => TTT (F)	67	68
	total	519	533
hydrophilic- stop	CAA (Q) => TAA (X)	2	2
	CAG (Q) => TAG (X)	2	2
	CGA (R) => TGA (X)	3	3
	total	7	7

The distribution of these sites across the PCGs, was highly conserved between the two species (**Figure 5**). The number of editing sites per gene varied significantly, ranging from a maximum of 44 sites in the *nad5* gene for both species, down to only a single site in *cox1*, also in both species. High numbers of editing events were consistently found in genes involved in NADH dehydrogenase, such as *nad5*, *nad7*, and *nad2*, as well as those for cytochrome c biogenesis, including *ccmB*, *ccmFn* and *ccmC*. Conversely, *cox1* possessed the fewest sites.

Further analysis of the nucleotide conversion types revealed that all RNA editing sites in both *C. kwangsiensis* and *C. longa* were canonical C-to-U conversions (**Supplementary Table 4**). This overwhelming prevalence of C-to-U editing is a hallmark of angiosperm mitogenomes and is essential for maintaining the functional integrity of mitochondrially-encoded proteins, often by restoring conserved amino acid identities at the protein level. In *C. longa*, the majority of edits occurred at the second codon position (427 sites, 64.0%), followed by the first codon position (241 sites, 36.0%). A virtually identical distribution was found in *C. kwangsiensis*, with 415 sites (63.7%) at the second position and 236 sites (36.3%) at the first position. Edits at the third codon position were negligible or absent in both species.

### Sequence divergence and selective pressure analysis

**Figure 6:**
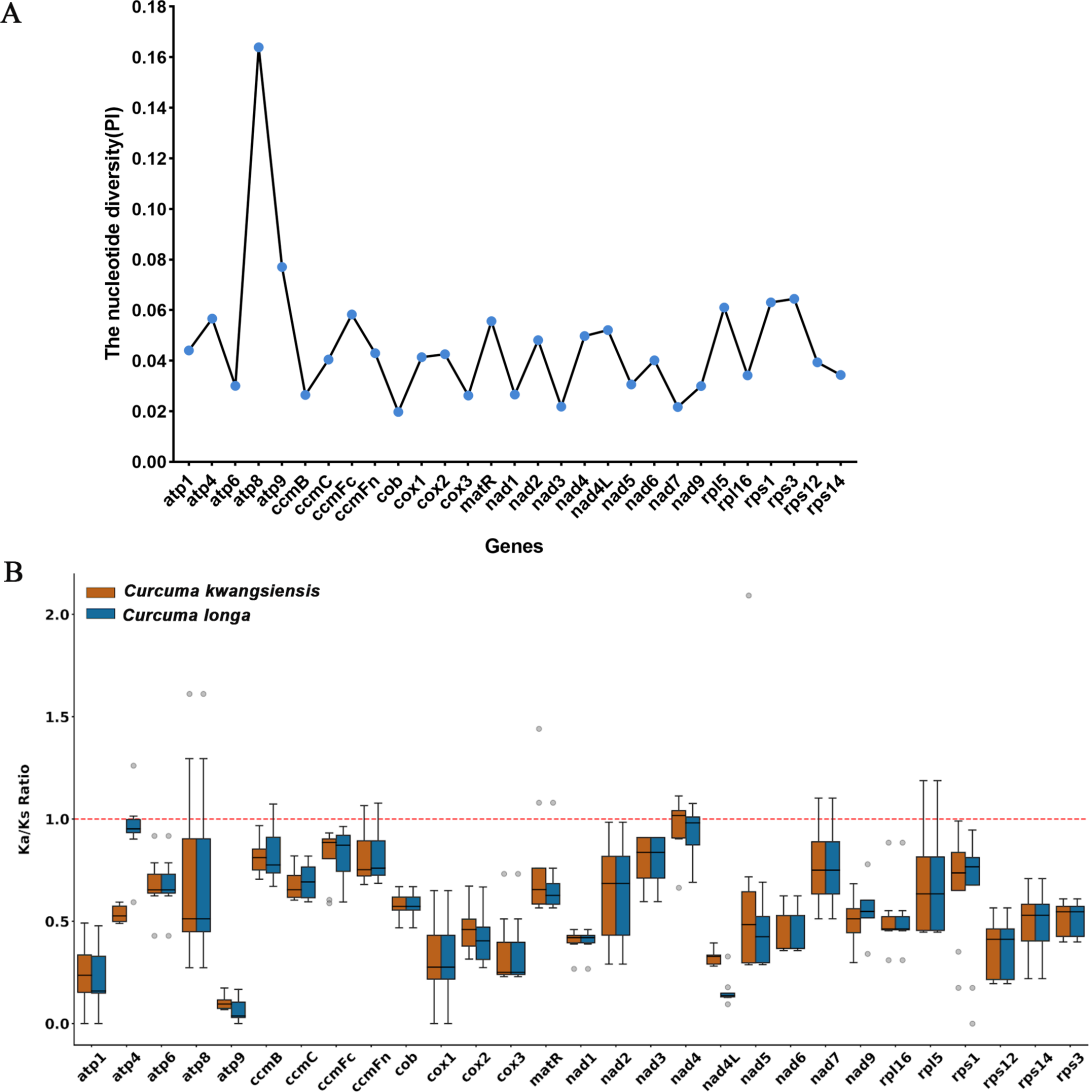
Nucleotide diversity (Pi) of conserved protein-coding genes **(A)** and distribution of Ka/Ks ratios for shared mitochondrial genes **(B)** in *C. kwangsiensis* and *C. longa* mitogenomes. The solid black line within each box indicates the median value. The dashed red horizontal line indicates the neutral selection threshold where Ka/Ks equals 1.

To evaluate the sequence divergence and evolutionary forces acting on the *Curcuma* mitogenomes, we first calculated the nucleotide diversity (Pi) for each of the 29 shared protein-coding genes between *C. kwangsiensis* and *C. longa* (**Figure 6A**). The analysis revealed a considerable range of divergence, with an overall mean Pi for all 29 genes of 0.0463. Divergence values varied substantially from gene to gene, indicating different evolutionary rates. The highest level of nucleotide diversity was observed in the *atp8* gene, which showed a Pi value of 0.1639. Conversely, the *cob* gene was the most conserved, exhibiting the lowest nucleotide diversity with a Pi value of only 0.0198.

To understand the selective pressures driving this divergence, we next calculated the non-synonymous (Ka) to synonymous (Ks) substitution ratios (Ka/Ks) for each gene against a range of related species. A total of 220 gene pairs were analyzed for *C*. *kwangsiensis* and 217 gene pairs were analyzed for *C*. *longa* (**Supplementary Table 5**). The evolutionary patterns were highly similar and conserved between the two *Curcuma* species (**Figure 6B**). The vast majority of gene pairs exhibited Ka/Ks ratios well below 1.0, indicating that these genes are predominantly under strong purifying negative selection. Specifically, in *C*. *kwangsiensis*, 205 of the 220 pairs, representing 93.2% of the total, showed Ka/Ks values less than 1. An almost identical proportion was found in *C*. *longa*, where 202 of 217 pairs, or 93.1%, were also under purifying selection. The overall median Ka/Ks value for *C*. *kwangsiensis* was 0.552, and the median for *C. longa* was likewise low at 0.565. Evidence for positive selection (Ka/Ks > 1) was rare, observed in only 15 individual gene pairs for each species. A detailed comparison revealed that these positively selected pairs were largely concentrated in the same set of genes for both species. Pairs in seven genes, *atp8*, *ccmFn*, *cox2*, *matR*, *nad4*, *nad7*, and *rpl5*, were found to be under positive selection in *both C. kwangsiensis* and *C. longa*. Beyond this substantial overlap, *C. kwangsiensis* possessed one unique gene, *nad5*, while *C*. *longa* showed two unique genes, *atp4* and *ccmB*, with evidence of positive selection. This pattern, with a strong overlap in positively selected genes, suggests similar, episodic adaptive pressures acting on specific lineages, even while the genes remain under strong purifying selection overall.

### Mitochondrial genome synteny

**Figure 7:**
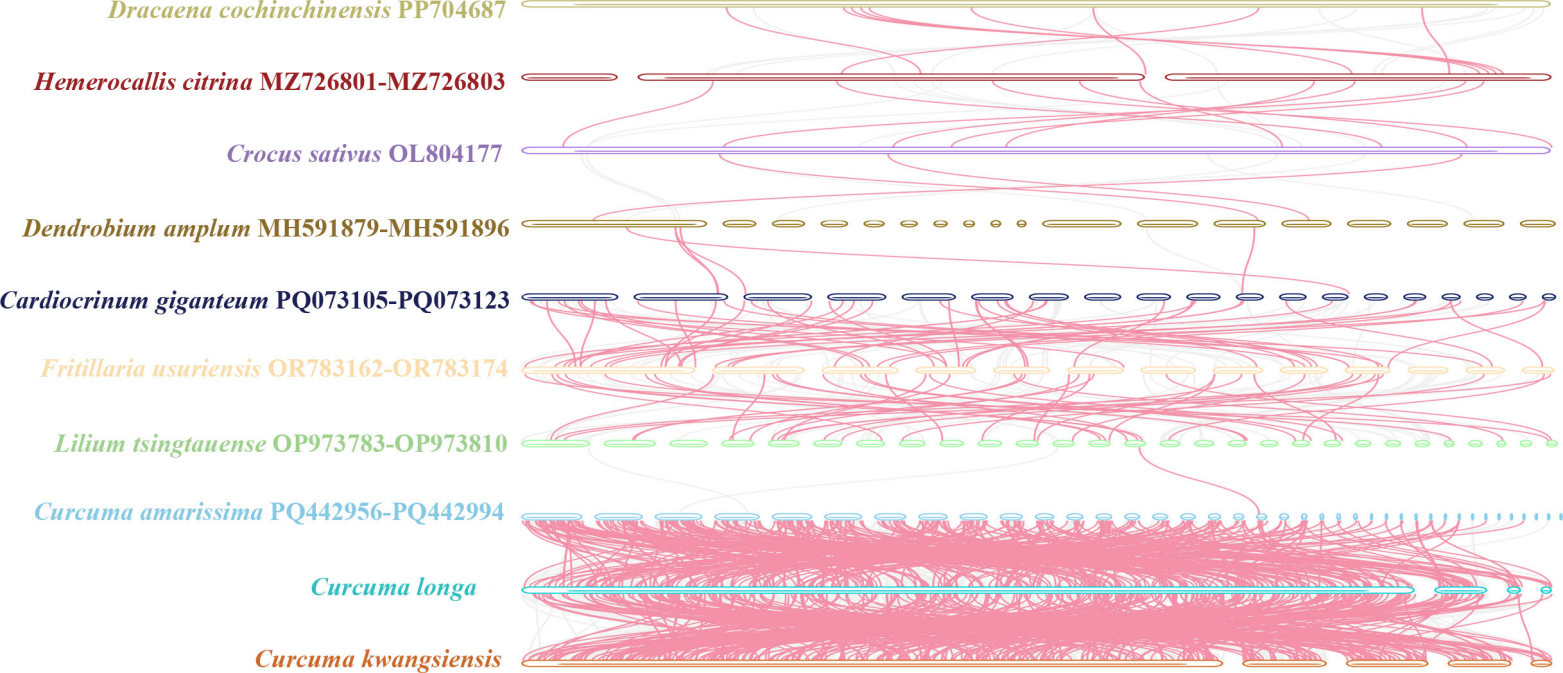
Whole-genome synteny analysis of *Curcuma* mitogenomes and related species. Each colored block represents a locally collinear block (LCB), indicating a region of shared homology. Lines connecting blocks illustrate the rearrangement of genomic regions. Blocks shown below the center line indicate an inverted orientation relative to the reference genome.

To comprehensively investigate structural evolution, we performed a whole-genome synteny analysis comparing our two newly sequenced mitogenomes, *C. longa* and *C. kwangsiensis*, against eight other representative species (**Figure 7**). The multi-species comparison revealed a highly dynamic genomic architecture across the entire group. When focusing specifically on the direct comparison between *C. longa* and *C. kwangsiensis*, this structural dynamism was particularly pronounced. The analysis confirmed a profound lack of collinearity between them. The relative order and orientation of locally collinear blocks (LCBs) are poorly conserved, demonstrating that their genomic structures have diverged significantly since their last common ancestor. While this macro-structure is highly fluid across the genus, we next quantified the micro-level sequence identity within the conserved syntenic blocks. A whole-genome BLASTn comparison between *C. longa* and *C. kwangsiensis* identified a total of 5,611,212 bp of homologous regions (**Supplementary Table 6**). Within these aligned regions, the weighted average sequence identity was 97.67%. This indicated that the *Curcuma* genus exhibits the characteristic dichotomy of angiosperm mitogenomes, where a remarkably fluid genomic structure that undergoes frequent rearrangement coexists with an extremely conserved primary sequence content.

### Gene transfer between mitogenome and chloroplast genome

**Figure 8:**
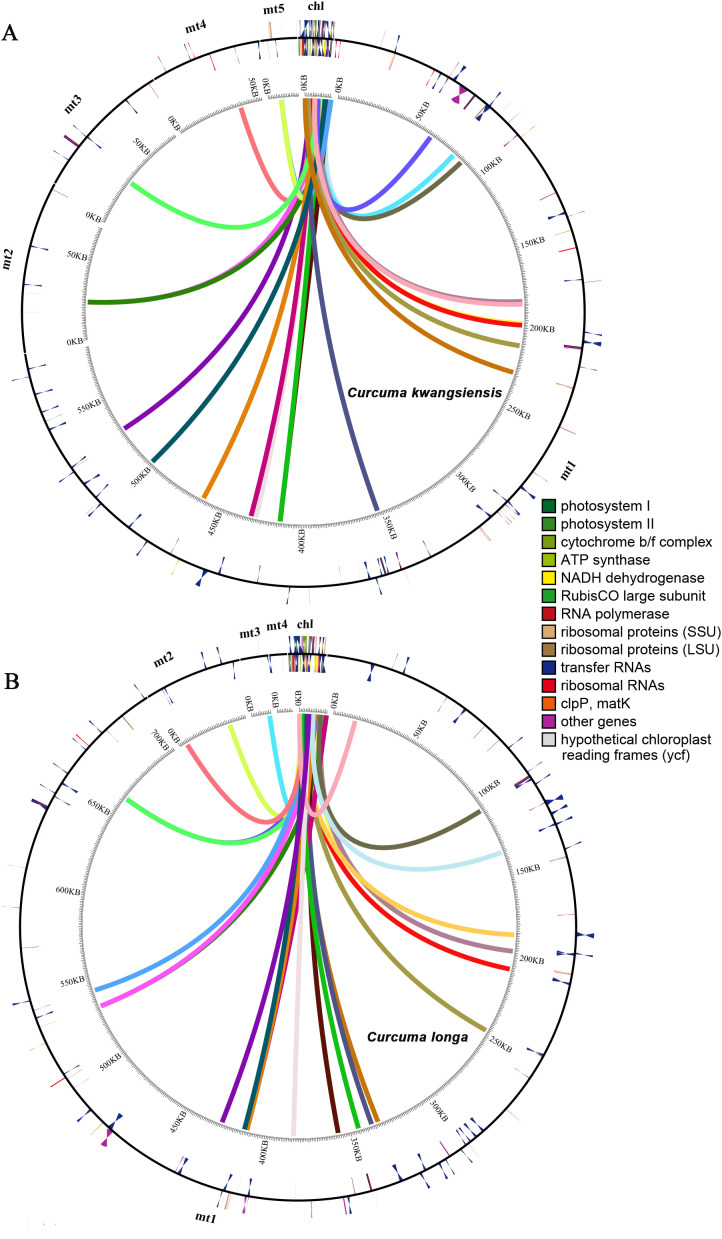
Homologous fragments transferred between the mitochondrial and chloroplast genomes in *C. kwangsiensis***(A)** and *C. longa***(B)**. Lines connect homologous regions larger than 1,000 bp, illustrating the extensive intracellular DNA transfer from the chloroplast to the mitochondrion.

Sequence similarity analysis identified 25 fragments in the *C. kwangsiensis* mitogenome that were homologous to its corresponding chloroplast genome, with an identical number of fragments found in *C. longa* (**Figure 8**). In *C. kwangsiensis*, the fragment lengths ranged from 1,011 bp to 7,378 bp, with a cumulative length of 59,103 bp, constituting approximately 0.74% of the overall mitogenome length (**Supplementary Table 7**). In *C. longa*, the length of homologous fragments was shorter than in *C*. *kwangsiensis*, with a cumulative length of 54,098 bp, ranging from 935 bp to 5,515 bp. By annotating these homologous sequences, we identified a large suite of transferred chloroplast-derived genes, revealing a dynamic state of gene capture and fragmentation. A total of 39 unique plastid genes were identified within the *C. kwangsiensis* fragments, of which 18 were transferred as intact genes and 21 were found only as partial gene fragments. A similar, yet distinct, profile was found in *C. longa*, which contained 41 unique plastid genes, with 20 genes transferred completely and 21 found as fragments. Several genes, such as *atpB*, *rpoB* and *trnI-GAU*, were found as complete copies in *C. kwangsiensis* but only as fragments in *C. longa*. Conversely, *rpl14* was complete in *C. longa* but fragmented in *C. kwangsiensis*. This indicates that the transfer of genetic material from the chloroplast to the mitochondrion is a complex and ongoing evolutionary process, followed by species-specific gene fragmentation and differential preservation.

### Phylogenetic reconstruction and evolutionary positioning

**Figure 9:**
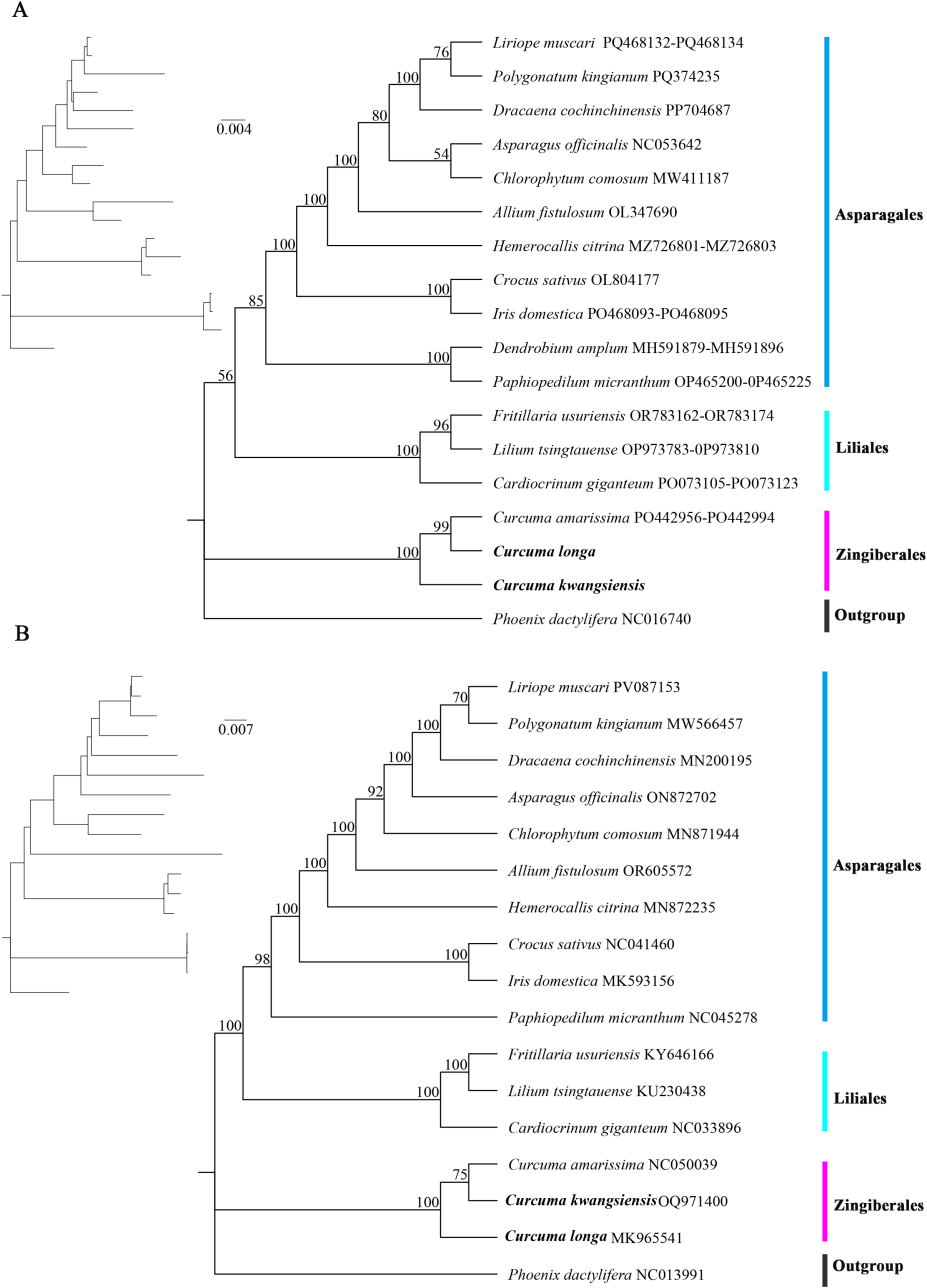
Phylogenetic reconstruction of *Curcuma* and related monocot species based on mitochondrial **(A)** and chloroplast **(B)** protein-coding genes. The Maximum Likelihood tree illustrates the evolutionary relationships among 18 species, with *Phoenix dactylifera* designated as the outgroup.

To elucidate the evolutionary relationships and phylogenetic positioning of *C. kwangsiensis* and *C. longa* within the broader context of monocotyledonous evolution, a Maximum Likelihood tree was reconstructed using 18 mitochondrial genomes and 17 chloroplast genomes (**Figure 9**). Both reconstructed topologies were highly robust. They accurately resolved the evolutionary lineages across several major orders including Asparagales, Liliales, and Zingiberales. The representative species from Asparagales and Liliales were organized into distinct, well-supported monophyletic clades, with the majority of critical nodes exhibiting the maximum bootstrap support of 100. *P. dactylifera* was strategically selected as the outgroup to root the monocot phylogeny.

In the mitochondrial tree (**Figure 9A**), within the genus *Curcuma*, the species clustered together to form a unified group with 100% bootstrap support. *C. longa* and *C. amarissima* were most closely related, sharing a sister-taxa relationship supported by a bootstrap value of 99%. *C. kwangsiensis* occupied a basal position relative to this sister pair within the *Curcuma* clade. The relatively short branch lengths and the scale bar indicating 0.004 substitutions per site further emphasized the high degree of sequence conservation within the core mitochondrial PCGs of these species. However, the chloroplast tree (**Figure 9B**) clustered *C. longa* and *C. kwangsiensis* together as sister taxa with 100% support. The chloroplast phylogeny showed a higher substitution rate with a scale bar of 0.007 substitutions per site. Taken together, the phylogenetic resolution from both organellar genomes provided a stable framework for understanding the divergence and species-specific evolutionary dynamics of the *Curcuma* genus within the Monocotyledonae.

## Discussion

Mitochondrial genomes in angiosperms are characterized by their remarkable structural plasticity, frequent recombination, and variable incorporation of foreign sequences, yet retain highly conserved coding content essential for energy metabolism (Gualberto and Newton, 2017; Zardoya, 2020). Within the Zingiberaceae, mitogenomic research remains scarce, with only a few species analyzed to date, limiting our understanding of genome evolution, structural dynamics, and phylogenetic relationships in this economically and medicinally important family (Liang et al., 2025). In this study, we reported the first complete mitochondrial genomes of *C. longa* and *C. kwangsiensis*, providing an unprecedented view of their genome architecture, gene content, codon usage, RNA editing landscape, and plastid-derived sequence incorporation. These data not only fill a critical knowledge gap for *Curcuma* mitogenomics but also provide a comparative framework to explore lineage-specific evolution, adaptive fine-tuning, and interspecies genomic divergence within the family. The findings have broad implications for understanding mitochondrial genome plasticity in Zingiberaceae, complementing prior studies on other monocot mitogenomes, and lay the foundation for integrative phylogenomic analyses incorporating nuclear and plastid datasets.

### Structural dynamics of *Curcuma* mitogenomes reveal unusually expanded architectures with conserved base composition

The complete mitochondrial genomes of *C*. *longa* and *C*. *kwangsiensis* presented a highly fragmented architecture, with total genome sizes of 7.66 Mb and 7.96 Mb, respectively. While these values fall within the broad range observed in certain monocotyledonous plants, including other members of the Zingiberaceae (Li et al., 2020), the inability to resolve these sequences into a single circular molecule was primarily a reflection of the inherent biological and computational complexity associated with such massive, repeat-rich structures. These large repeats frequently mediated intra- and inter-molecular recombination, resulting in a dynamic population of genomic isoforms that likely existed *in vivo* as branched or linear concatenated networks rather than a simple circle, a biological reality supported by the complex, interconnected paths visualized in our assembly graphs (**Supplementary Figure 1**). Furthermore, the presence of multiple branching points within the graph created significant path ambiguity. Consequently, maintaining the assembly as high-confidence scaffolds derived directly from the assembly graph provided a more scientifically rigorous representation of the *Curcuma* mitogenome. The overall GC content was nearly identical between the two species, at 43.90% and 43.69%, reflecting a conserved compositional bias. This combination of extreme genome expansion with compositional stability aligned with the characteristic dichotomy of plant mitogenomes, wherein sequence content was highly conserved while structural organization remains remarkably plastic (Oldenburg and Bendich, 2015).

Comparative synteny analysis revealed extensive disruption of collinearity between *C*. *longa* and *C*. *kwangsiensis*, as well as across other representative Zingiberaceae species. LCBs were poorly conserved in both order and orientation, illustrating the fluidity of mitogenome architecture even among closely related species (Kozik et al., 2019). Such structural dynamism has been reported widely across angiosperms, where frequent intramolecular recombination, repeat-mediated rearrangements, and horizontal incorporation of foreign DNA contribute to the mosaic nature of mitochondrial genomes (Najer et al., 2024). The observed fragmentation of key genes, such as *nad1* in *C*. *kwangsiensis*, further emphasizes that structural reorganization was an ongoing process, capable of generating species-specific mitogenomic configurations (Arrieta-Montiel and Mackenzie, 2011). Such extreme structural organization remains remarkably plastic while core sequence content remains relatively stable across divergent monocot lineages (Liu et al., 2014).

The processes underlying genome expansion and low collinearity in *Curcuma* seemed to be multifactorial, with repetitive sequences playing a central role. Our repeat analysis indicated that *C. kwangsiensis* exhibited a significantly more robust repeat landscape than *C. longa*, which likely contributed to its larger genome size. Dispersed repeats in *C. kwangsiensis* reached a total count of 9,322 on the first molecule alone, with a notable frequency peak of 3,059 repeats in the 300 to 349 bp length interval. Conversely, *C. longa* exhibited only 2,277 repeats within the same length category. These repeat elements created hotspots for intramolecular recombination, facilitating the duplication, inversion, and translocation of genomic segments (Gualberto and Newton, 2017). Tandem repeats enhanced the structural complexity of these mitogenomes. While tandem repeats in *C. longa* were predominantly restricted to the first molecule, they were found to be ubiquitous across all five molecules in *C. kwangsiensis*. The accumulation of these repeat sequences, alongside the horizontal incorporation of plastid-derived fragments and potential mobile element activity, provided the necessary sequence material for genome expansion without necessarily altering core coding capacity (Mower et al., 2012). Although large-scale validation was beyond the scope of this study, these repeats provide a foundation for developing molecular markers to evaluate *Curcuma* germplasm diversity. Collectively, the establishment of these superior mitochondrial genomic resources provided a crucial basis for investigating the multi-partite structure and adaptive evolution of medicinal plants in the Zingiberaceae family.

### Codon usage bias, extensive RNA editing, and purifying selection reinforce mitochondrial functional maintenance while enabling targeted adaptation

The analysis of codon usage revealed a significant preference for codons ending in A or U across both *Curcuma* mitogenomes, with leucine and serine being the most commonly encoded amino acids. This bias, quantified via RSCU values, probably reflected selection for translational efficiency and accuracy (Liu and Xue, 2005). Such patterns were optimized to match the usage of abundant tRNAs, thereby facilitating efficient protein synthesis across divergent plant lineages (Duret, 2002). The high similarity in codon usage patterns between *C. longa* and *C. kwangsiensis* suggested that selective pressures on translational optimization were widely conserved within the genus, maintaining the essential metabolic integrity of the mitochondrial machinery.

RNA editing represented another dimension of post-transcriptional regulation that reinforced mitochondrial function. Both species exhibited a high number of canonical C-to-U editing events, mostly in genes encoding membrane-associated respiratory proteins such as *nad* subunits and *ccm* components (Ichinose and Sugita, 2016). Notably, a large proportion of these edits resulted in the substitution of hydrophilic residues with hydrophobic ones, which was consistent with the structural requirements of membrane-spanning protein domains (Takenaka et al., 2013). These alterations likely enhanced protein stability and respiratory efficiency, offering a mechanism for fine-tuning mitochondrial function beyond genomic sequence alone (Castandet and Araya, 2012).

Selective pressure analysis further supported the functional conservation of *Curcuma* mitogenomes. A large number of PCGs exhibited Ka/Ks ratios well below 1.0, indicating strong purifying selection acting to maintain essential energy metabolism functions. A limited number of genes, including *atp8*, *ccmFn*, *cox2*, *matR*, *nad4*, *nad7*, and *rpl5*, exhibited evidence of episodic positive selection, suggesting that adaptive modifications had occurred in response to species-specific pressures while the overall mitochondrial machinery remained highly constrained (Mower et al., 2007). This pattern demonstrated the complex evolutionary mechanisms shaping plant mitogenomes as purifying selection acts to maintain essential functions, while rare and targeted positive selection permitted adaptation to fluctuating environmental conditions or metabolic requirements (Gao et al., 2014). Together, the combination of codon usage bias, RNA editing, and selective pressure illustrated how *Curcuma* mitogenomes balance functional maintenance with the capacity for targeted adaptation, ensuring both genomic stability and organismal fitness.

### Plastid-to-mitochondrion gene transfer and genomic rearrangements enhance mitogenome plasticity and species-specific evolutionary patterns

Horizontal gene transfer from the plastid to the mitochondrion significantly contributes to genome expansion and structural dynamism in *Curcuma* (Wang et al., 2007). Each species comprised 25 plastid-derived fragments, accounting for 0.7 to 0.8% of the mitogenome. These fragments included a combination of complete genes and partial sequences, exhibiting significant interspecies variation in the preservation of intact copies. For example, *atpB* and *rpoB* were preserved in full in *C*. *kwangsiensis* but appeared as fragmented sequences in *C*. *longa*, whereas *rpl14* was complete in *C. longa* but partial in *C*. *kwangsiensis*. Such species-specific variations highlighted that plastid-derived sequences were not merely passive genomic accumulations but were influenced by selective retention, degradation, and functional integration, hence shaping lineage-specific mitogenomic pathways (Sloan and Wu, 2014).

The phylogenetic reconstruction based on concatenated protein-coding genes (PCGs) offered a robust basis for understanding the maternal evolutionary positioning of *C. longa* and *C. kwangsiensis* within the Zingiberales clade. Plant mitochondrial sequences typically exhibit very low synonymous substitution rates. In this study, the rate was calculated at 0.004 substitutions per site. Traditional evolutionary models suggest that such conservativeness is highly effective for resolving deep-level phylogenetic relationships (Palmer and Herbon, 1988). This stability is beneficial because it avoids the saturation of substitution sites often found in faster-evolving markers. By utilizing a concatenated dataset of all available PCGs, we accumulated a sufficient number of informative sites to provide high statistical support (Bootstrap = 100), effectively overcoming the slow evolutionary rate of individual genes (Sloan et al., 2012). More importantly, the maternal inheritance of the mitogenome provides a clear evolutionary lineage that is significantly less susceptible to the confounding effects of hybridization and incomplete lineage sorting, which frequently occur in the nuclear genomes of the Zingiberaceae family (Mower et al., 2012). The contrast between highly conserved protein-coding sequences and a significant lack of genomic collinearity reflects a fundamental dichotomy in plant mitogenomes. While purifying selection maintains the sequence content, the overall structural organization remains remarkably plastic (Gualberto and Newton, 2017).

## Conclusion

In conclusion, this study provides a thorough comparative analysis of the complete mitogenomes of *C. longa* and *C. kwangsiensis*, revealing that core protein-coding genes remained highly conserved while the overall genomic architecture exhibited significant plasticity. Although sequence divergence in protein-coding regions was minimal, the two species were clearly distinguished by the differential retention of plastid-derived sequences and species-specific SSR patterns, such as the unique presence of a microsatellite in the *atp9* gene of *C. longa*. These findings demonstrated that horizontal gene transfer and extensive genomic rearrangements were the primary drivers of evolutionary dynamics within the *Curcuma* genus. Future research integrating multi-omic data will be crucial to comprehensively elucidate the complex evolutionary mechanisms and environmental adaptations of these important medicinal plants.

## Data Availability

The datasets presented in this study can be found in online repositories. The names of the repository/repositories and accession number(s) can be found in the article/**Supplementary Material**.

## Supplementary material

Supplementary Table 1 Annotation of protein-coding genes, transfer RNAs, and ribosomal RNAs in the mitogenomes of *C. kwangsiensis* and *C. longa*.

Supplementary Table 2 Codon usage and Relative Synonymous Codon Usage values for the protein-coding sequences of *C. kwangsiensis* and *C. longa*.

Supplementary Table 3 Comprehensive identification and genomic distribution of repetitive elements including dispersed repeats, tandem repeats, and simple sequence repeats in *C. kwangsiensis* and *C. longa* mitogenomes.

Supplementary Table 4 Detailed information of predicted RNA editing sites in the protein-coding genes of *C. kwangsiensis* and *C. longa* mitogenomes.

Supplementary Table 5 Nonsynonymous and synonymous substitution rates and Ka/Ks ratios for protein-coding genes in the mitogenomes of *C. kwangsiensis* and *C. longa*.

Supplementary Table 6 Comparative synteny analysis and identification of locally collinear blocks among the mitogenomes of *C. longa*, *C. kwangsiensis*, and *C. amarissima*.

Supplementary Table 7 Identification and characterization of plastid-derived DNA fragments in the mitogenomes of *C. kwangsiensis* and *C. longa*.

Supplementary Table 8 Sequencing data sources of *C. kwangsiensis* and *C. longa*.

Supplementary Figure 1 Visualized assembly graph of the *C. kwangsiensis* and *C. longa* mitogenomes.

Supplementary Figure 2 Sequencing depth distribution across the *C. kwangsiensis* and *C. longa* mitogenomes.
