# Supplementary figures and images for: Comparative analysis of complete mitochondrial genomes from Curcuma longa and Curcuma kwangsiensis reveals structural plasticity, conserved core genes, and species-specific evolutionary dynamics
Source: Front Plant Sci. 2026 Apr 15;17:1782424. doi: 10.3389/fpls.2026.1782424 (PMC13125149; doi:10.3389/fpls.2026.1782424)

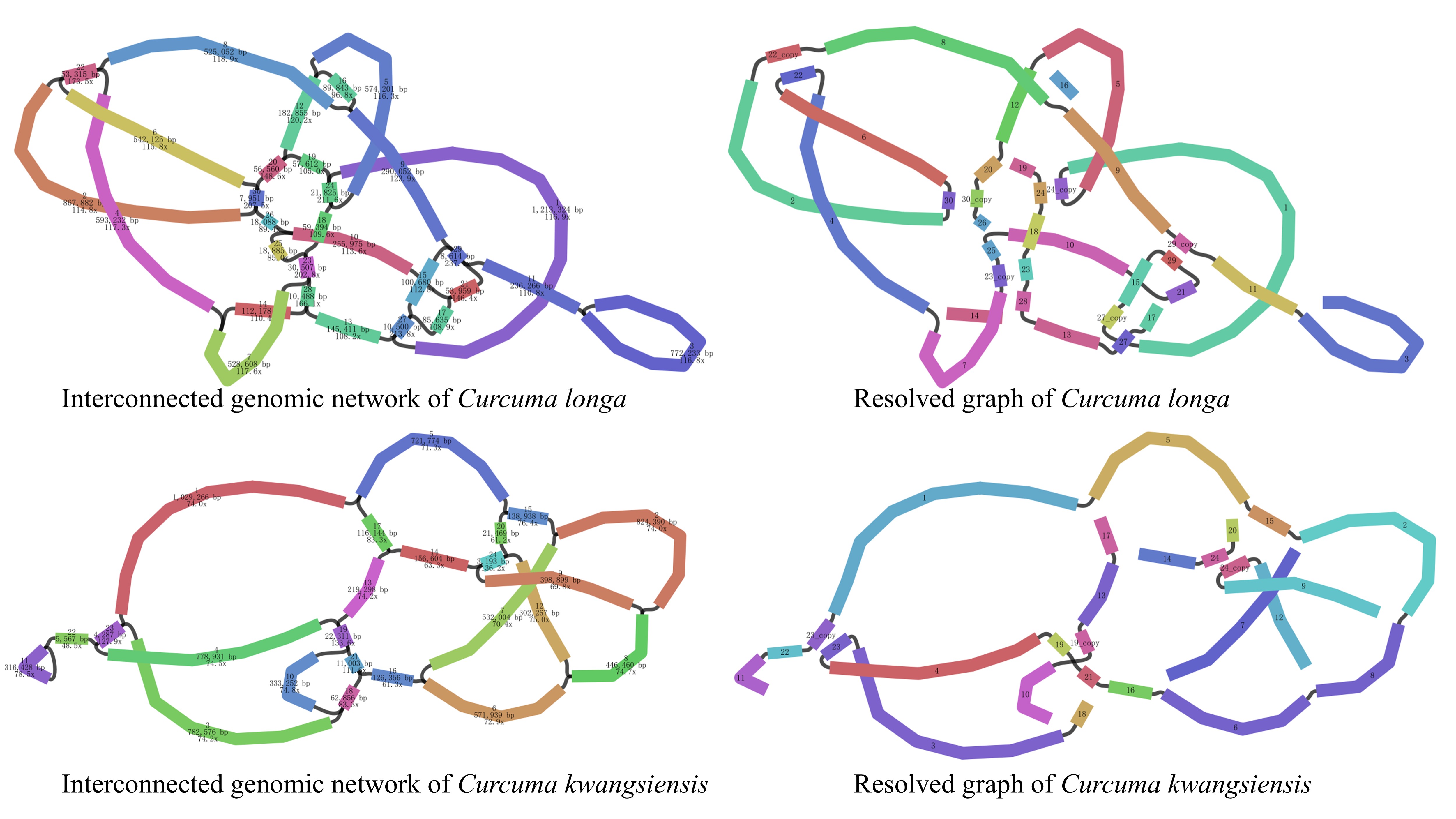

Supplement: Supplementary Figure 1 — Visualized assembly graph of the C. kwangsiensis and C. longa mitogenomes. [file Image1.jpeg]

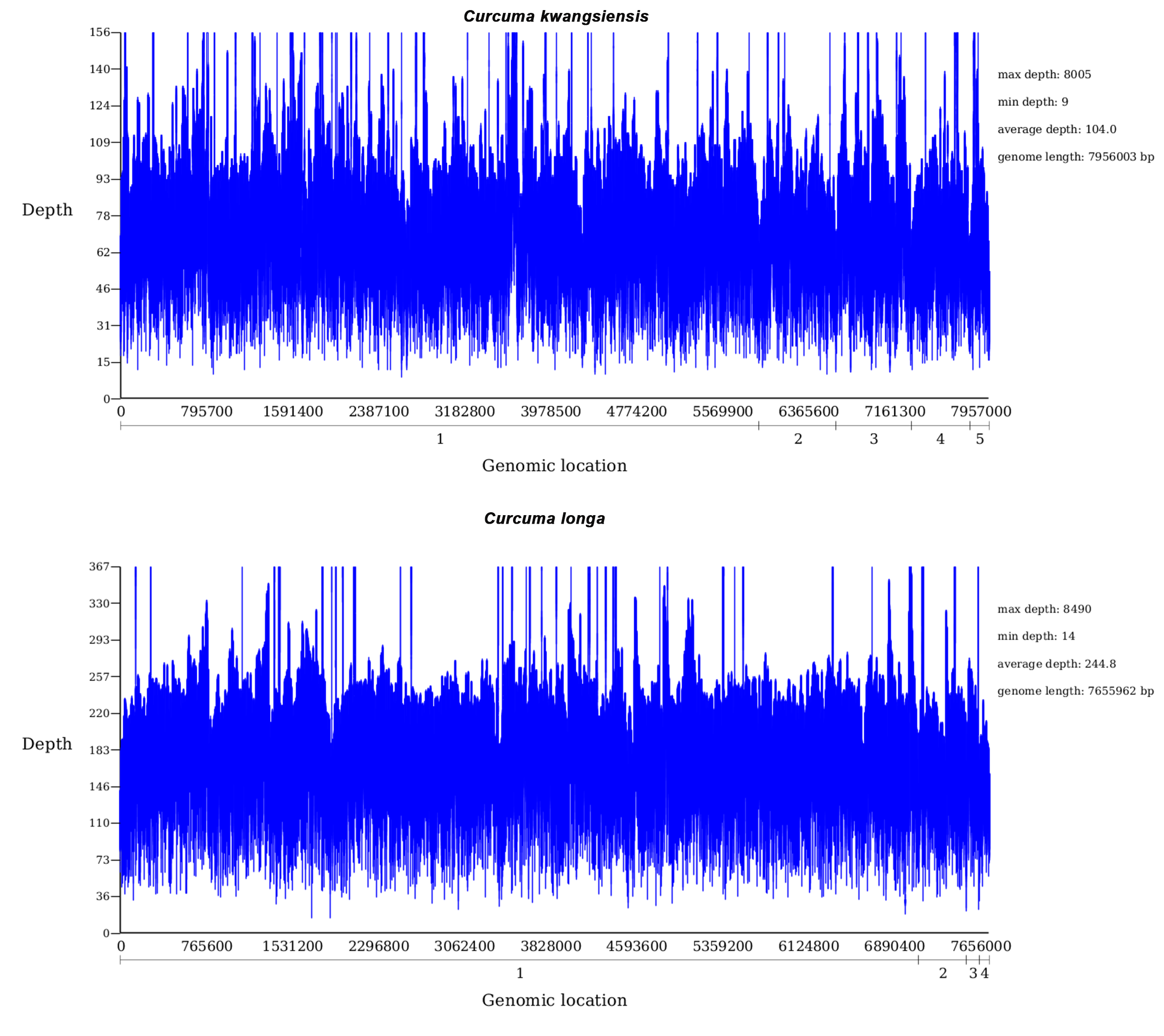

Supplement: Supplementary Figure 2 — Sequencing depth distribution across the C. kwangsiensis and C. longa mitogenomes. [file Image2.tif]
